# Identification and *in silico* structural analysis of *Gallus gallus* protein arginine methyltransferase 4 (PRMT4)

**DOI:** 10.1002/2211-5463.12323

**Published:** 2017-10-10

**Authors:** Hannah Berberich, Felix Terwesten, Sinja Rakow, Peeyush Sahu, Caroline Bouchard, Marion Meixner, Sjaak Philipsen, Peter Kolb, Uta‐Maria Bauer

**Affiliations:** ^1^ Institute of Molecular Biology and Tumor Research (IMT) Philipps‐University Marburg Germany; ^2^ Institute of Pharmaceutical Chemistry Philipps‐University Marburg Germany; ^3^ Department of Cell Biology Erasmus MC Rotterdam The Netherlands

**Keywords:** epigenetic regulation, histone arginine methylation, homology modeling, Pleckstrin homology domain, protein arginine methyltransferase 4, protein–protein docking

## Abstract

Protein arginine methyltransferase 4 (PRMT4) is an essential epigenetic regulator of fundamental and conserved processes during vertebrate development, such as pluripotency and differentiation. Surprisingly, PRMT4 homologs have been identified in nearly all vertebrate classes except the avian genome. This raises the possibility that in birds PRMT4 functions are taken over by other PRMT family members. Here, we reveal the existence of a *bona fide* PRMT4 homolog in the chicken, *Gallus gallus*. Using a biochemical approach, we initially purified a putative chicken PRMT4 protein and thus provided the first evidence for the presence of an endogenous PRMT4‐specific enzymatic activity toward histone H3 arginine 17 (H3R17) in avian cells. We then isolated a *G. gallus PRMT4* (gg*PRMT4*) transcript encompassing the complete open reading frame. Recombinant ggPRMT4 possesses intrinsic methyltransferase activity toward H3R17. CRISPR/Cas9‐mediated deletion of gg*PRMT4* demonstrated that the transcript identified here encodes avian PRMT4. Combining protein–protein docking and homology modeling based on published crystal structures of murine PRMT4, we found a strong structural similarity of the catalytic core domain between chicken and mammalian PRMT4. Strikingly, *in silico* structural comparison of the N‐terminal Pleckstrin homology (PH) domain of avian and murine PRMT4 identified strictly conserved amino acids that are involved in an interaction interface toward the catalytic core domain, facilitating for the first time a prediction of the relative spatial arrangement of these two domains. Our novel findings are particularly exciting in light of the essential function of the PH domain in substrate recognition and methylation by PRMT4.

**Abbreviations**
ADMA: asymmetric dimethyl arginine
CARM1: coactivator‐associated arginine methyltransferase 1
H3R17me2a: asymmetric dimethylation of histone H3 at arginine 17
MT: methyltransferase
PH: Pleckstrin homology
PRMT4: protein arginine methyltransferase 4
SAM: S‐adenosyl‐L‐methionine

Protein arginine methyltransferases (PRMTs) comprise an enzyme family that post‐translationally modifies a multitude of nuclear and cytoplasmic proteins. These enzymes transfer a methyl group from the ubiquitous cofactor S‐adenosyl‐L‐methionine (SAM) to the terminal guanidino nitrogens of arginine residues in their substrate proteins. Subsequent to the formation of monomethyl arginine (MMA) as an intermediate, type I PRMTs generate asymmetric (ω‐N^G^,ω‐N^G^) dimethyl arginine (ADMA), whereas type II enzymes give rise to symmetric (ω‐N^G^,ω‐N’^G^) dimethyl arginine (SDMA) 1. In mammals, nine PRMTs (PRMT1‐9) have been identified, which regulate a wide range of cellular functions, for example, signal transduction, nucleocytoplasmic transport, transcription, RNA processing, and DNA repair. Given the involvement of PRMTs in essential processes of eukaryotic physiology, the enzyme family and its modifications are believed to be ancient in evolution. Consistently, phylogenetic searches for PRMT homologs in nonmammalian animals revealed that PRMTs can be found in nearly all groups of eukaryotes. PRMT1 and PRMT5, which catalyze the majority of ADMA and SDMA production, respectively, are both present across every eukaryote species studied, whereas the distribution of other PRMT members seems to be restricted 2, 3. For example, PRMT4, also named CARM1 (coactivator‐associated arginine methyltransferase 1), was detected in most invertebrates with more than 70% sequence identity, but absent from the genome of nematodes. Furthermore, PRMT4 was found conserved with a high degree (>90%) of sequence identity in all vertebrate classes except the avian genome 2.

PRMT4 was the first PRMT member functionally linked to epigenetic regulation through asymmetric dimethylation of histone H3 4. Several transcription factors, such as steroid hormone receptors, STAT5 and c‐MYB, that interact with PRMT4 have been reported to place the enzyme close to chromatin and nucleosomal H3 1, 5, 6, 7. Subsequently, PRMT4‐mediated methylation of arginine 17, 26, and 42 in H3 promotes transcription. As an example, asymmetric dimethylation of H3R17 (H3R17me2a) is recognized by the TUDOR domain‐containing coactivator TDRD3 and furthermore facilitates chromatin recruitment of the transcription elongation‐associated complex PAF1 8, 9. Additionally, the nontail modification H3R42me2a destabilizes nucleosomes due to weakening of the interaction between the histone octamer and DNA 10. Apart from histone H3, PRMT4 modifies also other nuclear proteins, such as transcription factors, the coregulators p160 and CBP/p300, the mediator subunit MED12, and the C‐terminal domain of RNA polymerase II, thereby exerting transcriptional coactivating as well as corepressing functions 11, 12, 13, 14, 15, 16.

Mice lacking PRMT4 die perinatally due to lung dysfunction and exhibit further developmental defects compared to their wild‐type littermates 17, 18. Enzyme‐dead‐knockin mice show deficiencies similar to those seen in the knockout mice, indicating that the catalytic domain of PRMT4 is essential for most of its *in vivo* functions 19. Detailed analyses of the biological relevance of PRMT4 revealed that the enzyme is required for pluripotency and self‐renewal of stem cells and progenitor cells as well as for cell fate and differentiation decisions in various organs, such as the immune system, muscle, and adipose tissue 7, 20, 21, 22, 23, 24.

Consistent with reports showing that PRMT4 is highly expressed in immune cells and controls their differentiation as well as activation on gene expression level 25, we recently found that PRMT4 interacts with the transcription factor c‐MYB in human hematopoietic cells and coactivates c‐MYB‐dependent target gene expression in cooperation with the chromatin remodelers Mi2α/β 7. C‐MYB is a key regulator of vertebrate hematopoiesis and predominantly expressed in immature hematopoietic cells regulating the proliferation and differentiation of stem as well as progenitor cells 26. Initially, MYB was identified as a retroviral oncoprotein of avian leukemia viruses indicating its functional conservation in birds 27, 28.

Interestingly, we observed that in chicken macrophages c‐MYB‐dependent transcription is coactivated upon overexpression of mammalian PRMT4, suggesting that the enzyme and its function might have evolved together with the interaction partner c‐MYB and various substrates, such as H3R17, also in the avian lineage 7. Given that a BLAST search of the currently available *Gallus gallus* genome fails to identify a PRMT4 homolog, we raised the question whether avian PRMT4 exists. A biochemical approach enabled the isolation of the putative chicken ortholog on protein level and provided the first evidence for the presence of endogenous enzymatic activity of PRMT4 in avian cells. Sequence homology searches using the human *PRMT4* cDNA as query and a subsequent multistep cloning strategy resulted in the identification of a *G. gallus PRMT4* (gg*PRMT4*) transcript encompassing the complete ORF. The avian ortholog shows more than 90% sequence identity with human PRMT4 and possesses intrinsic catalytic activity toward H3R17. We used the published crystal structure of murine PRMT4 for protein modeling of chicken PRMT4. The overall high sequence identity between chicken and mammalian PRMT4 leads to a predicted high structural similarity, yet with avian‐specific variations in the Pleckstrin homology (PH) domain. *In silico* analyses of the relative spatial arrangement of the PH and the catalytic domain provided for the first time a prediction of the interaction interface between these two domains. Strictly conserved amino acids within the PH domain of birds and other vertebrates are responsible for this interface establishing the structural basis for the essential catalytic functions of the PH domain.

## Materials and methods

### Cell lines

HD11 chicken macrophages were maintained in Iscove's modified Dulbecco's medium (Gibco, Thermo Scientific) supplemented with 8% FBS (Gibco) and 2% chicken serum (Sigma Aldrich) at 37 °C and 5% CO_2_. Sf9 cells were cultured in Sf‐900™ II SFM medium (Gibco) and maintained at 27 °C and 90 r.p.m.

### RNA isolation, cDNA synthesis, and PCR amplification

Total RNA from HD11 cells was isolated using the PeqGold total RNA Kit (PeqLab). For first‐strand cDNA synthesis, 500 μm oligo(dT)_17_ primers was annealed to 2 μg of total RNA at 70 °C for 10 min prior to the addition of reaction buffer (20 mm Tris pH 8.5, 50 mm KCl, 10 mm DTT, 500 μm dNTPs, and 25 mm MgCl_2_). Following 5 min of incubation at 42 °C, 200 U SuperScript II reverse transcriptase (Thermo Scientific) and 20 U RiboLock RNase inhibitor (Thermo Scientific) were added and incubated for 90 min at 42 °C. After inactivation at 70 °C for 15 min, RNA was digested using 5 U RNase H (NEB) for 20 min at 37 °C. Subsequently, 0.75 μL of cDNA was subjected to PCR amplification using 1 U Phusion polymerase (Thermo Scientific), 500 nm of various PRMT4 homology primers (listed below), 500 μm dNTPs, and 2% DMSO. To maximize the PCR product yield, touch‐down PCR was applied with annealing temperatures ranging from 64 °C to 55 °C in addition to the standard Phusion polymerase program. The following primers were employed for cDNA amplification:

**Table:** 

Forward ggEST#1	5′‐GCCAACGAGAGAGTCCAAC‐3′
Forward ggEST #2	5′‐TTCCAGTTCTACGGGTACCTCTC‐3′
Forward ggEST #3	5′‐AATCGTCGGCTGTGCAGT‐3′
Forward ggEST #4	5′‐CGCCAAGAAGTACCTCAAGC‐3′
Reverse ggEST	5′‐GGCCAAATCCGTCAAATACACC‐3′
Forward human homology primer	5′‐ATCTAAGATGGCAGCGGCG‐3′
Reverse reptile homology primer	5′‐TTAGCTCCCATAATGCATTGTGT‐3′

### Identification and amplification of ggESTs and full‐length ggPRMT4 transcript

Human *PRMT4* mRNA (NCBI ref seq: NM_199141.1) was used as a query sequence for a Nucleotide Basic Local Alignment Search (BLAST, NCBI) of the *G. gallus* expressed sequence tag (EST) database. Therefore, two cDNA clones ChEST394e4 and ChEST665c21 were identified. To receive additional sequence information of the 5′‐ and 3′‐end of the putative gg*PRMT4* transcript, a forward primer encompassing the start codon of human *PRMT4* was combined in touch‐down PCR with the reverse primer that binds the 3′‐end of the previously identified *G. gallus* cDNA fragment. Therefore, an additional 74‐bp fragment was identified from the cDNA of HD11 cells, which included a start codon and 22 bp of the 5′‐UTR sequence of the putative chicken *PRMT4* ortholog. To obtain also the 3′‐end sequence information of gg*PRMT4* mRNA, various reptile cDNA sequences were extracted from the NCBI nucleotide database and subjected to multiple sequence alignments with the MUSCLE tool. Based on the most frequent nucleotides at each position, a reptile homology reverse primer was designed. This reverse primer was applied together with a forward primer encompassing the ATG of gg*PRMT4* and resulted in an amplicon of 2000 bp from HD11 cDNA providing the remaining 450 bp of coding sequence of the 3′‐end including the stop codon and an additional 220‐bp sequence of the 3′‐UTR. Accuracy of the resulting full‐length coding sequence of the chicken *PRMT4* transcript was confirmed by three independent rounds of RNA isolation, cDNA amplification, and Sanger sequencing (LGC Genomics) of both strands. The complete *G. gallus PRMT4* transcript has been deposited in the GenBank database with accession number KY655811.

### siRNAs

Small interfering RNAs (siRNAs) were purchased from Dharmacon or Eurogentec. The sequences of the control siRNAs were provided by Dharmacon. The sense strands of the various siRNAs are indicated below.

**Table:** 

siCtrl.1	5′‐ AUGAACGUGAAUUGCUCAA ‐3′
siCtrl.2	5′‐ UGGUUUACAUGUCGACUAA‐3′
siEST.1	5′‐ GCUGUGCAGUACUUCCAGU ‐3′
siEST.2	5′‐ UCAUCAUCUCGGAGCCCAU ‐3′

### Plasmids and clonings

The following plasmids were used for baculoviral expression in Sf9 cells: pFASTBAC‐flag‐mmPRMT4 was published by 12. The complete ORF of gg*PRMT4* (aa 1‐580) was amplified by mutagenesis PCR (forward primer: 5′‐TATAGAATTCATGGCGGCGGTG‐3′, reverse primer: 5′‐GACCCTCGAGTCAGCTGCCGTAGTGC‐3′) and inserted via *Eco*RI and *Xho*I into the pFASTBAC HT A vector (Invitrogen). Further, this *Eco*RI/*Xho*I fragment of gg*PRMT4* cDNA was cloned into pGEX4T1 vector (Sigma Aldrich) for expression of GST‐tagged full‐length ggPRMT4 in *E. coli*. The plasmid encoding GST‐tagged full‐length mmPRMT4 was published in 7. The plasmid pCMV‐Tag2‐flag‐rPRMT4 29 was used for overexpression of mammalian PRMT4 in HD11 cells.

The following target sites in gg*PRMT4* or GFP as control were chosen for guide RNA (gRNA) design:

**Table:** 

ggPRMT4_gRNA_1 (minus strand)	5′‐GCATTCGGTGTCGCGCGACA‐3′
ggPRMT4_gRNA_4 (plus strand)	5′‐TGCGCCTCGACGTCCGCGCC‐3′
ggPRMT4_gRNA_6 (plus strand)	5′‐CTCACCATCGGGGACGCCAA‐3′
GFP_gRNA	5′‐GGAGCGCACCATCTTCTTCA‐3′

Pairs of oligos for these targeting sites (including the PAM sequence) were annealed and cloned into BsmBI‐restricted lentiCRISPRv1 plasmid (Addgene), which enables bicistronic expression of Cas9 and gRNA.

### Transfections and infections

6 × 10^6^ HD11 cells per 15‐cm dish were transiently transfected with 50 μg plasmids using a standard CaPO_4_ transfection protocol. For siRNA transfections, HD11 cells (2.4 × 10^6^ cells per 10‐cm dish) were transfected with 20 μm siRNA using Metafectene Pro (Biontex) according to the manufacturer's protocol.

For CRISPR/Cas9‐mediated deletion of gg*PRMT4* in HD11 cells, HEK293T cells were transfected with the two packaging plasmids pMD2.G and psPAX2 together with the lentiviral expression plasmid lentiCRISPRv1 encoding the gRNA/controls and Cas9. Transfections were performed using X‐tremeGENE (Roche). Supernatants containing lentiviral particles were harvested one and 2 days after transfection and concentrated using PEG. For infection, 1.5 × 10^6^ HD11 cells per 10‐cm dish were seeded. At the next day, cells were infected in the presence of polybrene (8 μg·mL^−1^) with viruses encoding either the combination of all three gg*PRMT4* gRNAs or the GFP control gRNA. Cells were selected using puromycin and after single cell cloning maintained in the presence of 1 μg·mL^−1^ puromycin.

Recombinant baculoviruses were generated according to the Bac‐to‐Bac baculovirus system (Invitrogen). After one round of virus amplification, 20 × 10^6^ Sf9 insect cells per 15‐cm dish were infected with 7.5 μL virus/10^6^ cells.

### Antibodies

The following antibodies were used in western blot, immunofluorescence staining, and immunoprecipitation: Rabbit affinity‐purified anti‐murine PRMT4 was produced using His‐tagged recombinant protein corresponding to aa 433‐608 of murine PRMT4 7, anti‐human PRMT4 (09‐818, Merck Millipore, epitope aa 595‐608), anti‐β‐tubulin (MAB3408, Merck Millipore), anti‐H3R17me2a (ab8284, Abcam), anti‐H3 (ab1791, Abcam), anti‐ADMA (13522, Cell Signaling), anti‐Flag (F 3165, Sigma Aldrich), and anti‐rabbit IgG (I5006‐10MG, Sigma Aldrich).

### Immunofluorescence staining

HD11 cells (1.3 x 10^5^ /24 well) were plated on cover slips. After 24 h, cells were rinsed in PBS and fixed in methanol for 10 min at −20 °C. Subsequently, cells were washed in PBS, permeabilized in PBS/0.3% Triton X‐100 for 5 min, and blocked in PBS/4% BSA for 45 min. Then, cells were stained with the indicated antibodies in the presence of PBS/4% BSA for 2 h at room temperature. Afterward, cells were rinsed three times in PBS and stained with the secondary antibody anti‐rabbit Cy3 (Jackson Immuno Research) for 1 h at room temperature in the presence of 0.14 μg·mL^−1^ DAPI (4′,6‐diamidino‐2′‐phenylindole‐dihydrochloride) for nuclear/DNA staining. After the final washes in PBS, cells were mounted (Mowiol containing 25 mg·mL^−1^ DAPCO) and analyzed by fluorescence microscopy (Axioskop 2, Zeiss).

### Immunoprecipitation

For whole‐cell extracts (WCE), Flag‐PRMT4‐transfected and wt HD11 cells were washed with ice‐cold PBS and lysed in IPH buffer (50 mm Tris/HCl pH 8.0, 200 mm NaCl, 0.5% NP‐40, 5 mm EDTA, protease inhibitors) followed by treatment with 62.5 U Benzonase (Invitrogen) per mg protein lysate in the presence of 7.5 mm MgCl_2_ for 1 h at 4 °C to digest genomic DNA. Extracts were cleared by centrifugation. For subsequent immunoprecipitation, 1 mg WCE per IP was adjusted to 150 mm NaCl in a total volume of 1 mL. The extracts were incubated with 4 μg of the indicated antibodies overnight at 4 °C and then BSA‐blocked protein G agarose (GE Healthcare) was added for 2 h at 4 °C. The bead‐bound precipitates were subjected to extensive washes using cold IPH buffer and finally employed in western blot or methyltransferase (MT) assays.

### Recombinant protein preparation

GST‐tagged proteins were purified from *E. coli* BL21, eluted from glutathione Sepharose (GE Healthcare) in the presence of 50 mm Tris pH 8.0 including 25 mm reduced glutathione and finally dialyzed (PBS, 10% glycerol) using standard protocols. For protein preparation of recombinant Flag‐tagged mmPRMT4 and His‐tagged ggPRMT4, baculovirus‐infected Sf9 cells were washed twice with PBS prior to 3× freeze and thaw lysis in BC buffer (20 mm HEPES pH 7.9, 250 mm NaCl, 10% glycerin, 0.4 mm EDTA, 1 mm β‐mercaptoethanol, and 1 mm PMSF). Protein purification was performed using Ni‐NTA Sepharose (Qiagen) and anti‐Flag M2 Affinity Gel (Sigma Aldrich) as previously described 30. The concentration of recombinant proteins was determined by SDS/PAGE and Coomassie staining.

### Methyltransferase (MT) assays

For *in vitro* methyltransferase (MT) assays, 4‐16 μg of either bulk histones from calf thymus (Sigma Aldrich), purified core histones from calf thymus (Roche) or H3 peptides (aa 1‐25 amino acids synthesized by Peptide Specialty Laboratories Heidelberg, Germany) was incubated with precipitates from HD11 cells (bead‐bound PRMT4) or 1–2 μg of purified recombinant GST‐, Flag‐, or His‐tagged PRMT4 and 2 μL [^14^C‐methyl]‐SAM (58.3 nCi mm ^−1^, Perkin Elmer) in PBS for 3–12 h at 30–37 °C. Subsequently, the reactions were separated by SDS/PAGE, blotted, and analyzed by autoradiography.

### *In silico* model building

The protein–protein docking was performed using ZDock 3.0.2 31. The crystal structures 2OQB and 3B3F were used as input structures (Table S1). PDB ID 3B3F was chosen because it comprises two homodimers, which could be selected as one docking partner without altering the PDB file beforehand, to facilitate a maximally unbiased docking. To introduce random variations in the docking, four calculations were performed. In those calculations, the combinations of the chains of both input structures were permutated (3B3F [AB/CD], 2OQB [A/B]). A binding pose comprising nice shape complementarity and recurring in all four dockings within the first six solutions was chosen for further modeling.

Homology modeling was performed using MODELLER 9v14 32. For modeling the PH domain of PRMT4, the homolog from *Mus musculus* (PDB ID 2OQB) was used. The cofactor‐ and substrate‐binding domains were also modeled using PRMT4 from *M. musculus* (PDB ID 3B3J). Prior to model building, target and template sequences were aligned using MODELLER. For further refinement, the model for the PH domain was chosen according to the discrete optimized protein energy (DOPE) score. For the cofactor‐ and substrate‐binding domains, one model was duplicated and combined with the model of the dimer afterward.

The modeled domains were aligned to the results of the docking in PyMOL 33 and thereafter protonated and minimized in MOE 34, followed by an optimization of the docking poses using the Docking2 Rosetta Protocol with the ‘docking‐local‐refine’ option enabled 35, 36, 37. The model was then subjected to a 50‐ns molecular dynamics simulation using Amber 14 38 utilizing the ff99SB force field 39 to allow for larger‐scale relaxation of the relative orientation, followed by a final short geometry minimization for bond lengths, bond angles, and planarity using Phenix 1.10.1‐2155 40. The final model was evaluated with WHAT_IF 41 version WHATCHECK 7.0 and PROCHECK v.3.5 42. Ramachandran plot outliers were, where possible, manually corrected altering the dihedrals of the corresponding residues in *Coot* 43. The final Ramachandran plot is shown in Fig. S3.

## Results and Discussion

### Detection of endogenous PRMT4 protein and enzymatic activity in avian cells

**Figure 1:**
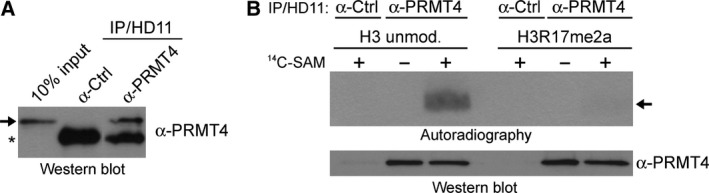
Detection of endogenous PRMT4 protein and catalytic activity in chicken cells. (A) Whole‐cell extracts from HD11 cells (1 mg) were subjected to immunoprecipitation (IP) of endogenous, putative chicken PRMT4 protein using antibodies specific for murine PRMT4 (α‐PRMT4, 7). IPs using isotype control IgG (α‐Ctrl) were performed as negative control in parallel. Input (10%) and IP reactions were analyzed by western blot using a commercial human PRMT4 antibody (Merck Millipore). The arrow indicates the 68‐kDa putative PRMT4 protein band in chicken cells. The asterisk marks the IgG heavy chain. (B) IPs from HD11 cells were performed using antibodies specific for murine PRMT4 (α‐PRMT4) and isotype control IgG (α‐Ctrl), as described in A. Precipitates were subjected to *in vitro* methyltransferase (MT) assays (overnight, at 30 °C) in the presence of either unmodified or R17me2a‐premodified H3 peptides (aa 1‐25) and in the absence (−) or presence (+) of ^14^C‐labeled SAM. Methylation products were resolved by SDS/PAGE, blotted, and analyzed by autoradiography (upper panel). The arrow indicates the histone H3 peptide band. Immunostaining of the blot with PRMT4 antibodies (α‐PRMT4) visualizes the bead‐bound PRMT4 used in the methylation assay as an input control (lower panel).

Recent observations indicate that c‐MYB‐dependent transcription is coactivated by PRMT4 in mammalian as well as avian cells 7, suggesting that the enzyme and its function are evolutionarily conserved also in the bird lineage. As PRMT4 has been found in all vertebrate classes with the exception of birds 2, we searched for the presence of endogenous enzymatic activity of PRMT4 in chicken cells. In a first step, we used antibodies generated in our laboratory against murine PRMT4 7 and performed immunoprecipitations from extracts of HD11 cells, a chicken macrophage cell line. Input and precipitates were immunoblotted with a second commercially available anti‐human PRMT4 antibody, which displayed the recognition of a 68‐kDa protein band, that is, within the expected molecular weight range of a putative chicken PRMT4 ortholog (Fig. 1A). Control antibodies did not precipitate this protein band (Fig. 1A). Next, we employed these precipitates in *in vitro* methyltransferase (MT) assays using ^14^C‐radiolabeled SAM and either unmodified histone H3 peptides or premodified at R17 (R17me2a). By autoradiography, we detected an enzymatic activity specific for H3R17 in chicken, as the anti‐PRMT4 precipitates exhibited methylation activity toward the unmodified but not premodified peptides (Fig. 1B). Control precipitates did not show any detectable enzymatic activity (Fig. 1B). H3R17 is a nonredundant and major methylation target of mammalian PRMT4 5, 44. As a control, we immunoprecipitated overexpressed Flag‐tagged murine PRMT4 from HD11 cell extracts and utilized the Flag precipitates in *in vitro* MT assays showing R17 methylation (Fig. S1). Collectively, these initial results provided the first experimental evidence for the existence of PRMT4 protein and activity in avian cells.

### Identification of expressed sequence tags (ESTs) in Gallus gallus with sequence similarity to human PRMT4

**Figure 2:**
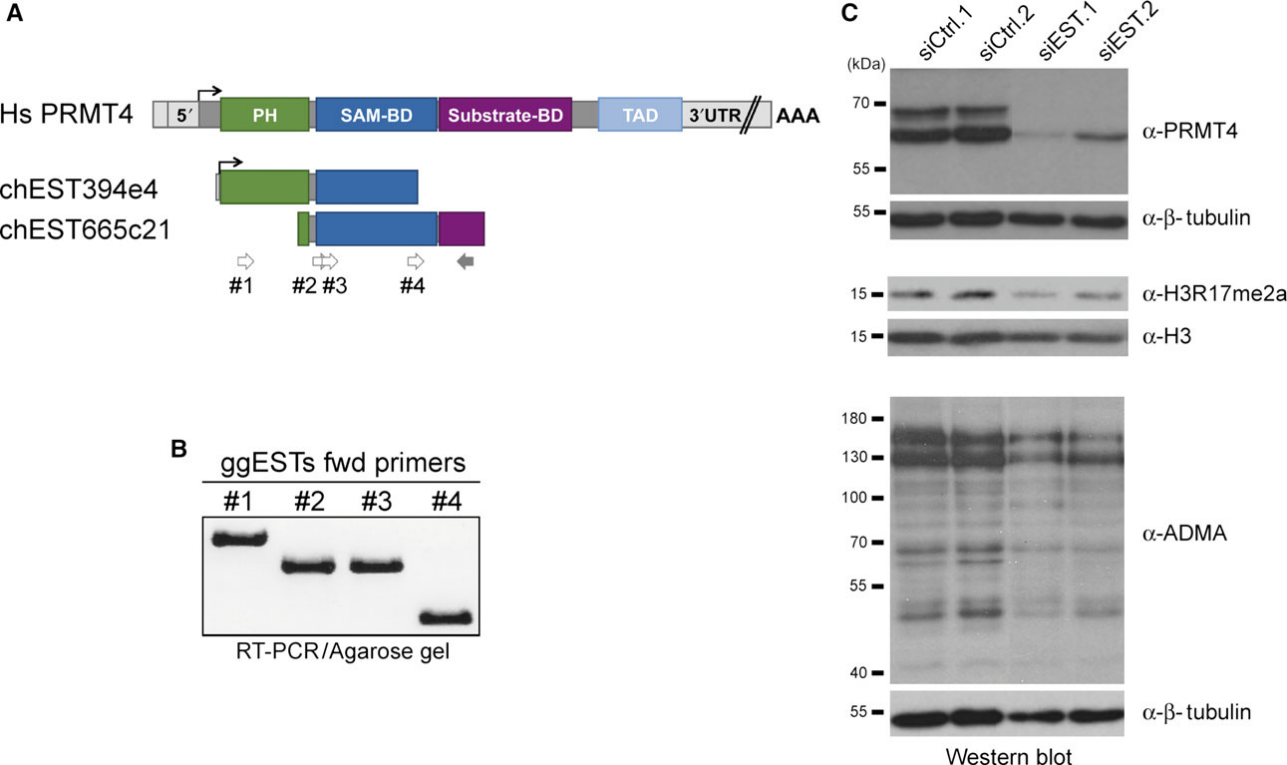
Identification of expressed sequence tags (ESTs) in *Gallus gallus* with high homology to human *PRMT4* and encoding the expected catalytic activity in chicken cells. (A) Schematic presentation of the *Homo sapiens PRMT4* mRNA and two partially overlapping *G. gallus* expressed sequence tags (ESTs), which were identified by nucleotide BLAST search using human *PRMT4* mRNA as query sequence. Black arrows indicate the translation start site, and colored boxes mark segments that translate into functional domains. Abbreviation of the functional domains: PH: Pleckstrin homology domain (green); SAM‐BD: S‐adenosyl‐methionine‐binding domain (dark blue); substrate‐BD: substrate‐binding domain (purple); TAD: transactivation domain (light blue). PCR primers used for amplification of the EST sequences from *G. gallus* cDNA (in B) are illustrated by gray arrows (bright gray: forward primers no. 1–4, dark gray: reverse primer). (B) Total RNA from HD11 cells was reverse‐transcribed by oligo(dT) priming and subsequently amplified by standard touch‐down PCR using the indicated forward primers in combination with the constant reverse primer (illustrated in A). PCR products were separated via agarose gel electrophoresis, excised, purified, and subjected to Sanger nucleotide sequence analysis. (C) HD11 cells were transfected with either nontargeting control siRNAs (siCtrl.1 and siCtrl.2) or two alternative siRNAs derived from the *G. gallus* EST sequences (in A) targeting putative chicken PRMT4 (siEST.1 and siEST.2). 72 h post‐transfection, whole‐cell extracts were prepared and analyzed by western blot using the indicated antibodies (α‐PRMT4, α‐H3R17me2a and α‐ADMA). Immunostaining with antibodies against histone H3 and β‐tubulin served as loading controls. Molecular weights of the protein marker bands are indicated on the left.

In the next step, the human *PRMT4* cDNA sequence (NM_199141.1) was employed as query sequence to search the *G. gallus* expressed sequence tag (EST) database for the putative chicken ortholog. Two partial chicken ESTs (chEST394e4 and chEST665c21) were found that exhibit significant sequence homology (83–85% identity) to the human *PRMT4* transcript and encoded the putative PH (Pleckstrin homology) domain, SAM‐binding domain and N‐terminal part of the substrate‐binding domain (Fig. 2A). As these ESTs overlapped across a 500‐bp segment sharing a sequence identity of 98%, we assumed that they derive from a single gene. For further investigation, we performed reverse transcription (RT)‐PCR employing RNA isolated from HD11 cells and several forward primers (Fig. 2A, #1 ‐ #4), complementary to both ESTs or only the 5′‐end of chEST394e4, in combination with a reverse primer complementary to the 3′‐end of chEST665c21 (Fig. 2A). The resulting amplicons showed the expected sizes, in particular also an approximately 1000‐bp PCR fragment spanning both ESTs (Fig. 2B, #1), indicating that the ESTs indeed originate from a single gene. Sanger sequencing analysis of these amplicons verified the EST sequences and their homology to the human *PRMT4* transcript. Next, we designed two alternative siRNA molecules (siEST.1 and siEST.2) based on this partial mRNA and transfected them into HD11 cells. Compared to control siRNA transfections, both siESTs resulted in reduced western blot detection of the 68‐kDa putative PRMT4 protein band by antibodies recognizing human PRMT4 (Fig. 2C). Furthermore, the levels of H3R17me2a and ADMA decreased upon siEST transfection in the chicken cell extracts (Fig. 2C). These results identified ESTs in *G. gallus* with high sequence similarity to human PRMT4 and confirmed the connection between the predicted *PRMT4* transcript and the putative PRMT4 protein in avian cells.

### Cloning of the complete CDS and parts of the UTR sequences of Gallus gallus PRMT4

**Figure 3:**
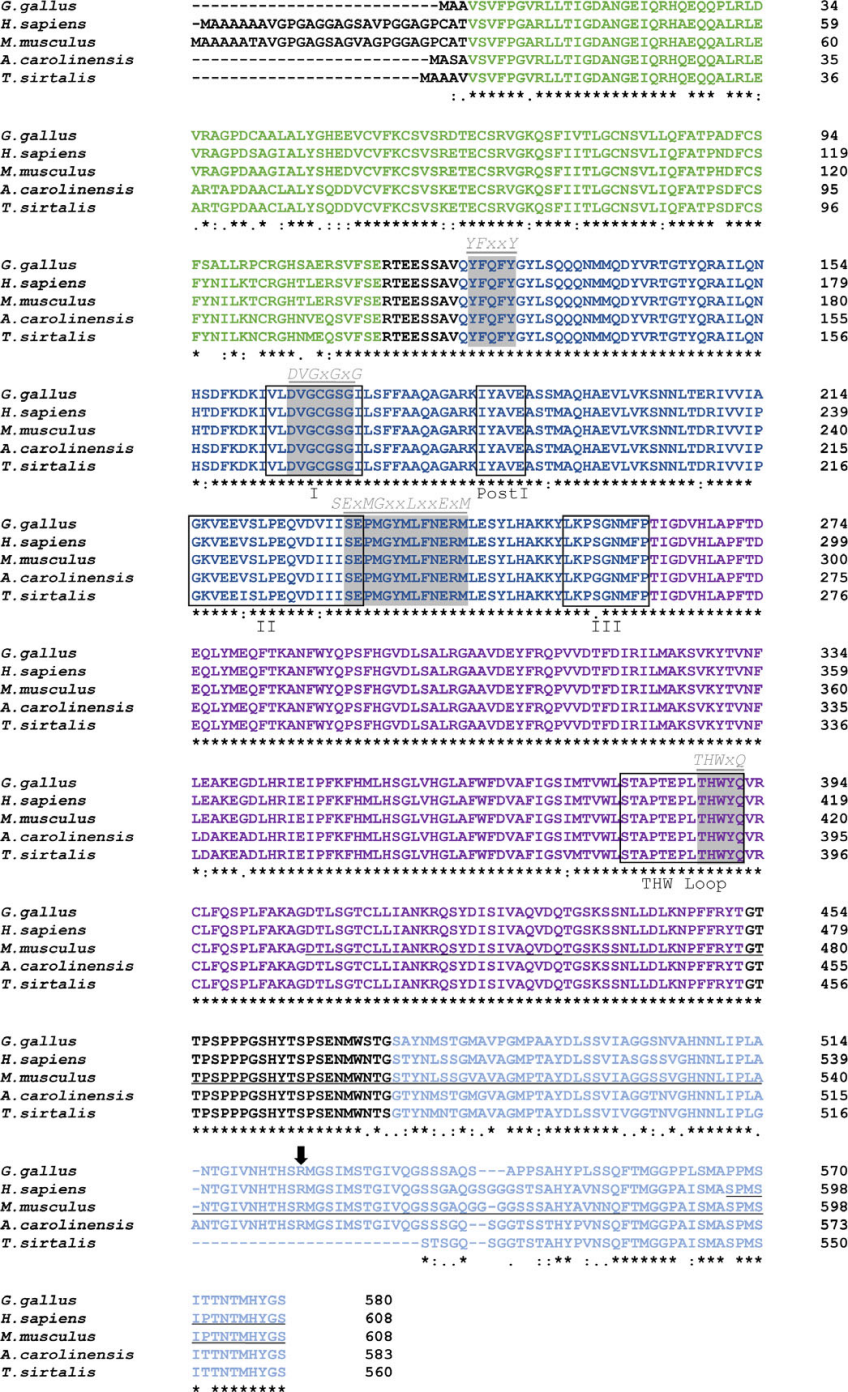
Amino acid sequence alignment of *Gallus gallus* PRMT4 and several vertebrate PRMT4 orthologs. The nucleotide sequence of *G. gallus PRMT4* was translated using ExPASy translation tool and aligned with multiple vertebrate PRMT4 protein sequences using Clustal Omega. Functional domains are highlighted (analogously to the color code in Fig. 2A) as follows: Pleckstrin homology domain (green), S‐adenosyl‐methionine‐binding domain (dark blue), substrate‐binding domain (purple), and transactivation domain (light blue). The consensus of the four signature sequences is underlined and written above. Rectangles encompass the conserved methyltransferase motifs I, Post I, II, III, and the THW loop, which partially overlap with the signature sequences. The arrow marks the conserved arginine residue for PRMT4 automethylation. Residue numbering is shown on the right of the sequence. Asterisks mark fully conserved amino acid residues. Colons indicate amino acid residues containing functional groups with strongly similar properties, while periods mark amino acids with weakly similar features. Underlined amino acids in the human (aa 595‐608) and murine (aa 433‐608) sequence indicate the epitopes of the anti‐mammalian PRMT4 antibodies employed in this study. The accession numbers for the protein sequences used in this alignment are as follows: *G. gallus* KY655811, *Homo sapiens* NP_954592.1, *Mus musculus* NP_067506.2, *Anolis carolinensis* XP_008102027.1, *Thamnophis sirtalis* XP_013913272.1.

To receive additional sequence information of the 5′‐ and 3′‐end of the putative gg*PRMT4* transcript, a multistep cloning strategy was employed utilizing homology primers derived from humans as well as reptiles (the latter as the phylogenetically closest relatives of birds). This approach resulted in the isolation of a *G. gallus PRMT4* (gg*PRMT4*) transcript encompassing the complete coding sequence, which has not been annotated in the currently available *G. gallus* genome (Ensembl Gallus_gallus‐5.0, last updated/patched‐Dec 2016). The gg*PRMT4* mRNA is 1743 bp in length and encodes a 580‐amino acid protein (Fig. 3). The corresponding protein shares more than 90% sequence identity with human PRMT4. Likewise, the epitopes of the antibodies recognizing murine and human PRMT4, which were employed in the detection of putative chicken PRMT4 protein (Fig. 1), are highly conserved in ggPRMT4 (Fig. 3). In agreement, these mammalian‐specific PRMT4 antibodies detected recombinant chicken PRMT4 proteins (Fig. S2). Alignment of ggPRMT4 and several representative vertebrate PRMT4 proteins revealed a high sequence conservation in the catalytic core domain and particularly in the four PRMT signature motifs of the cofactor‐ and substrate‐binding module, which are identical in sequence for the presented vertebrate species (Fig. 3, gray boxes). Similarly, the methyltransferase motifs I, Post I, II, III, and the THW loop are highly conserved. Outside the catalytic core, significant diversity was observed in the N‐ and C‐terminal sequences. For example, the first 25 amino acids, which have been predicted to be highly disordered in murine PRMT4, are specific for the mammalian homologs and are lost in the avian as well as in reptile lineage (Fig. 3), suggesting that this region might not contribute to essential functions of PRMT4 45. In contrast, the N‐terminal Pleckstrin homology (PH) domain and the C‐terminal transactivation domain (TAD), which are unique for PRMT4 within the PRMT family, are found with some sequence variations also in the avian homolog. Both domains are required for coactivator function and substrate specificity of PRMT4 46, 47. As an example, the automethylation site R548 within the TAD is strictly conserved among all vertebrate species including birds, indicating an evolutionary preserved and essential function of this residue for self‐regulation of PRMT4 in pre‐mRNA splicing and transcriptional activation 48. Altogether, these results identify the complete open reading frame (ORF) of *G. gallus PRMT4* with high sequence similarity to other vertebrate homologs.

### Intrinsic methyltransferase activity of recombinant Gallus gallus PRMT4 toward histone H3R17 and other cellular proteins

**Figure 4:**
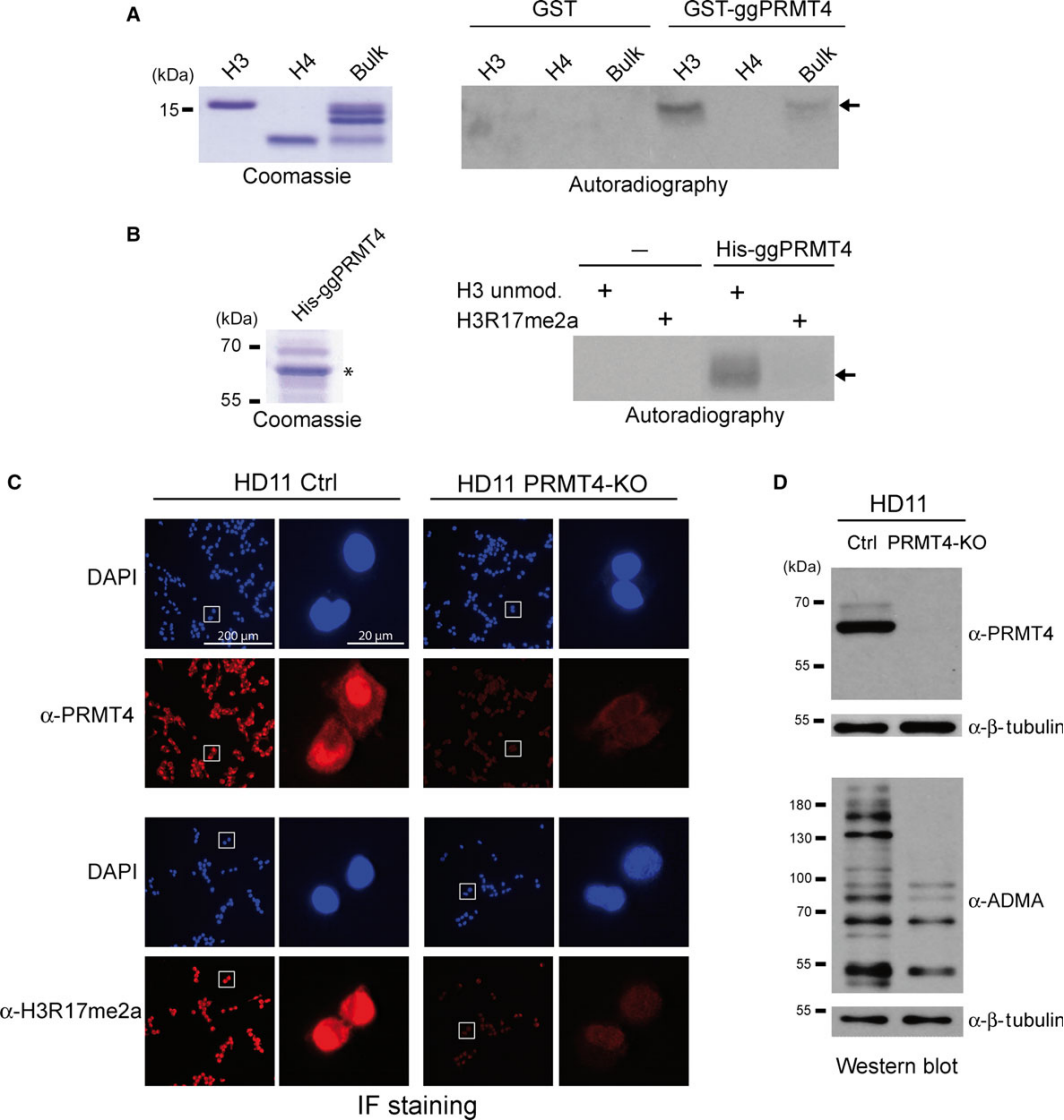
The *Gallus gallus* PRMT4 homolog is an arginine methyltransferase with substrate specificity for H3R17 *in vitro* and *in vivo*. (A) Purified core histones H3 and H4 from calf thymus as well as bulk histones from calf thymus, as visualized in the Coomassie Blue‐stained SDS gel (left panel, molecular weight of the 15‐kDa protein marker band is indicated), were subjected to *in vitro* MT assays in the presence of purified, eluted GST protein alone, or GST‐ggPRMT4 and ^14^C‐labeled SAM. Methylation products were resolved on SDS/PAGE, blotted, and visualized by autoradiography (right panel). The arrow indicates the histone H3 protein band. (B) Histone H3 peptides (aa 1‐25, unmodified or R17me2a) were subjected to *in vitro* MT assays in the absence (−) or presence (+) of recombinant His‐tagged ggPRMT4 and ^14^C‐labeled SAM. Coomassie Blue‐stained SDS gel visualizes the baculoviral expressed, purified, and eluted His‐tagged ggPRMT4 (left panel, asterisk marks the PRMT4 protein band). Molecular weights of the protein marker bands are indicated on the left. Methylation reactions were separated by SDS/PAGE, blotted, and visualized by autoradiography (right panel). The arrow indicates the histone H3 peptide band. (C) CRISPR/Cas9‐mediated PRMT4‐knockout (PRMT4‐KO) and control (Ctrl) HD11 cells were analyzed by immunofluorescence (IF) staining for the levels and distribution of PRMT4 (α‐PRMT4, upper panels) and histone H3R17 dimethylation (α‐H3R17me2a, lower panels). The corresponding DNA/nuclear stainings with DAPI are shown above. Right pictures represent higher magnifications of the framed areas of the left pictures. All images were taken with the same exposure time. Scale bars indicate 200 μm and 20 μm, respectively. (D) Whole‐cell lysates of PRMT4‐knockout (PRMT4‐KO) and control (Ctrl) HD11 cells (as in C) were prepared and analyzed by western blot using the indicated antibodies (α‐PRMT4 and α‐ADMA). Immunostainings with β‐tubulin antibodies served as loading control. Molecular weights of the protein marker bands are indicated on the left.

To confirm that the newly obtained ORF codes for the avian PRMT4 ortholog, we expressed GST‐tagged full‐length ggPRMT4 in bacteria and assayed the purified fusion protein for methyltransferase activity using purified histones H3 and H4 as well as bulk histones as substrates. These *in vitro* MT assays demonstrated that the recombinant protein intrinsically possesses catalytic activity and methylates specifically histone H3, but no other core histone, whereas GST alone did not show any methyltransferase activity (Fig. 4A). Given that bacterially expressed GST‐PRMTs are less active, in particular on peptide substrates, than recombinant PRMTs purified from insect cells (our own observation, data not shown), we established the baculovirus‐mediated expression in Sf9 cells and purification of His‐tagged full‐length ggPRMT4. This His‐tagged ggPRMT4 revealed that the enzyme specifically methylates R17 in histone H3, the well‐known methylation site of mammalian PRMT4 (Fig. 4B). To finally show that the gene, from which this newly identified *PRMT4* transcript derives, is responsible for the catalytic activity in chicken cells, we designed guide RNAs targeting the gg*PRMT4* coding sequence (within the PH domain), as no information on the genomic location of *PRMT4* is available due to its hitherto missing annotation in the *G. gallus* genome. Consequential CRISPR/Cas9‐mediated deletion of gg*PRMT4* in HD11 cells resulted in a complete loss of nuclear PRMT4 as well as H3R17me2a detection by immunofluorescence staining compared to control cells (Fig. 4C). Furthermore, the *in vivo* activity of the avian homolog was verified by the global loss of arginine‐methylated proteins in PRMT4‐knockout compared to control HD11 cells, as examined by western blot using ADMA antibodies (Fig. 4D). These results unambiguously show that the transcript identified here encodes a catalytically active arginine methyltransferase with the substrate specificity of PRMT4 and eventually confirms the existence of a PRMT4 ortholog in the bird lineage.

### *In silico* modeling of the three‐dimensional protein structure of Gallus gallus PRMT4

While the sequences of the cofactor‐ and substrate‐binding domains of PRMT4 are almost identical among the vertebrate species*,* the N terminus encompassing the PH domain and the C terminus differ to some extent in their sequences between the vertebrate homologs (Fig. 3). Given that the PH domain has recently been found to be responsible for substrate recognition and methylation of most PRMT4 substrates in human cells 49, we investigated here the sequence variations and conservations of chicken versus other vertebrate PH domains to elucidate its structural connection to the catalytic core domain and how this might translate to its essential enzymatic functions.

Although several crystal structures revealed the dimeric arrangement of the central catalytic domain of PRMT4, which is a structure commonly adopted by all type I PRMTs, the full‐length PRMT4 protein has not been crystallized yet. In the only X‐ray structure published to date based on murine PRMT4 protein encompassing aa 28‐507, the N‐terminal part (aa 28‐140) was disordered and therefore not visible (PDB ID 3B3J) 45. Interestingly, the isolated N‐terminal domain (PRMT4_28‐140_) displayed a PH domain fold (2OQB), which is known to mediate protein–protein interactions and to bind proline‐rich sequences 45. However, the structural arrangement of this functionally essential domain relative to the entire PRMT4 dimer is still enigmatic. Therefore, we used the respective crystal structures from *M. musculus* and combined protein–protein docking and homology modeling to first dock the PH domains to the crystal structure of the murine PRMT4 dimer and subsequently build a homology model of the ggPRMT4 dimer including the PH domains.

**Figure 5:**
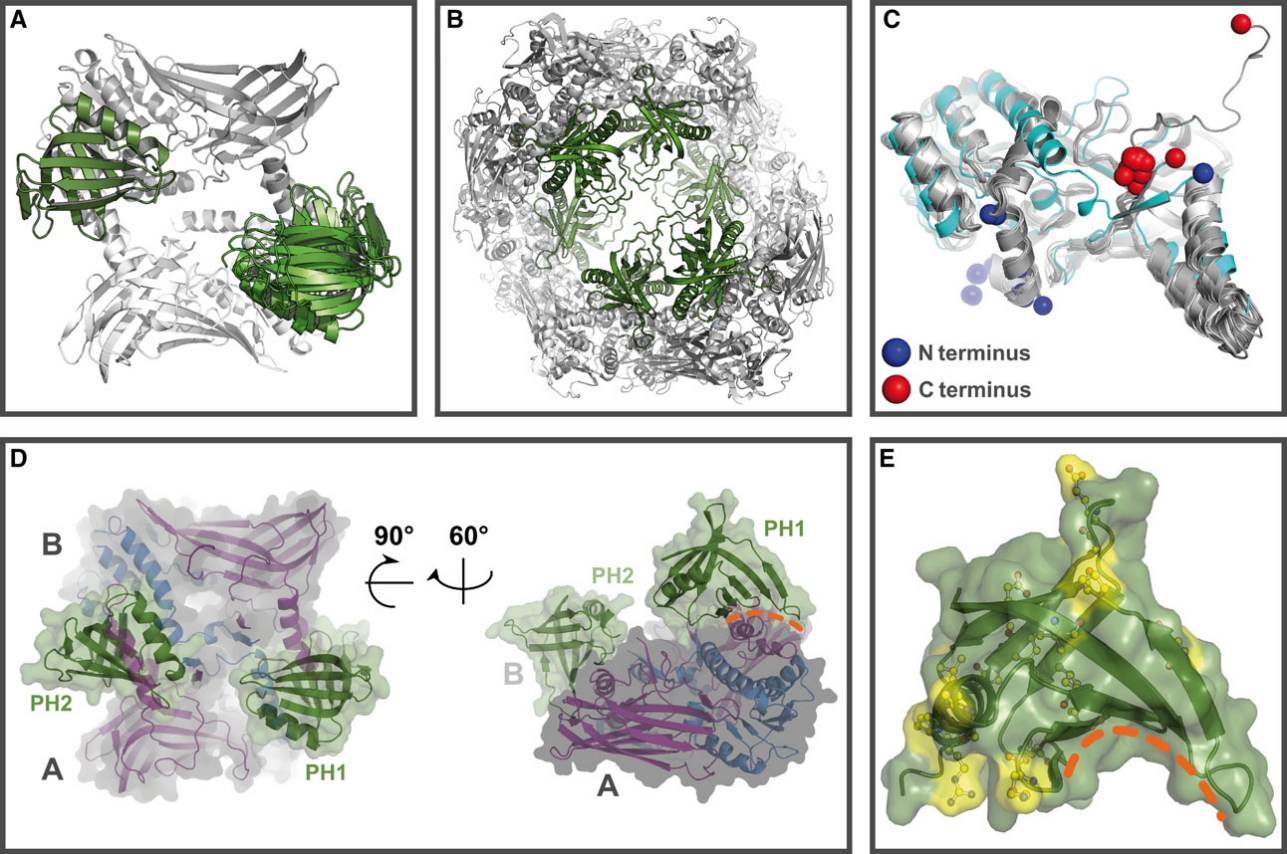
*In silico* modeling of the three‐dimensional protein structure of *Gallus gallus* PRMT4. (A) Top view of the docking poses, which shows the consensus docking pose selected for further modeling. The homodimer of the SAM‐ and substrate‐binding domains is shown in gray; the docking poses of the PH domains are shown in different shades of green. (B) Top view of the double helix formed by mmPRMT4 homodimers (gray) in the crystal structure of PDB ID 3B3J, generated by expanding the visualization in accordance with the hexagonal space group of the crystal. The PH domain from PDB ID 2OQB (green) is modeled using the binding poses resulting from the docking experiment. (C) Alignment of all 36 PRMT4 crystal structures deposited in the PDB archive until now (Table S1). Crystal structure 3B3J is highlighted in cyan. The differing positioning of the N (blue sphere) and C termini (red spheres) is easily discernible. In this context, the terms N terminus and C terminus refer to the last amino acid crystallographically resolved at the corresponding end of the protein sequence used. 3B3J is the only structure in which N terminus and C terminus are located on the same side of the protein. (D) Final model of the ggPRMT4 homodimer showing the spatial arrangement of the PH domains and the catalytic core domains (encompassing the SAM‐ and substrate‐binding domains). The domains are colored as follows: PH domain (green), SAM‐binding domain (blue), and substrate‐binding domain (purple). The homodimer including the PH domains is shown in a top view in the left picture. The side view orientation in the right picture is generated by rotating the first structure by 90° out of the paper plane followed by an approx. 60° rotation counterclockwise. In this depiction, the interface between the PH domain and the catalytic core domain is highlighted with a dashed orange line. (E) The sequence differences for the PH domain of PRMT4 between *Mus musculus* (2OQB) and *G. gallus* (homology model) are illustrated in the modeled ggPH domain. The orientation is chosen in accordance with the orientation of the PH domain in the right picture of 5D. Sequence differences are highlighted in yellow on the surface. In addition, the corresponding residues are shown in a yellow ball‐and‐stick representation. The conserved amino acids of the putative interface are marked with a dashed orange line.

In the first step, the mmPH domain was docked to the crystal structure of the catalytic core of mmPRMT4. Of the 36 existing crystal structures of PRMT4 in the PDB archive, we used the structure of mmPRMT4 with ID 3B3F, as it contains two homodimers in the unit cell, which can be selected as docking targets (Table S1). In four independent, unbiased protein–protein docking calculations, a biophysically possible binding pose could be found among the ten highest‐ranked docking solutions, and all four of these poses were similar to each other. This consensus pose occurred on the top ranks (namely two, four, four, and six, respectively) in each of the docking calculations. These four final docking poses are shown as an overlay in Fig. 5A. Interestingly, the PH domains cover the dimerization arms of the substrate‐binding domains, which is consistent with unhindered access to the substrate‐ and cofactor‐binding pockets. Furthermore, although the PH domain was not restrained to bind to a specific region of the dimer of the cofactor‐ and substrate‐binding domains, the C‐terminal residue of the PH domain in the model is located in proximity to the N‐terminal amino acid visible in the X‐ray structure 3B3J 45, consistent with a connection between them.

Inspecting the crystal structure 3B3J in more detail by expanding the visualization in accordance with the hexagonal space group P 6_2_ 2 2, it is striking how well the PH domain in its docking pose occupies the empty space in the protein crystal of mmPRMT4 (Fig. 5B). Residues 28‐140 were not resolved in X‐ray structure 3B3J 45, but the arrangement of the remaining domains and the packing of the protein in the crystal can be regarded as a negative imprint of the location and conformation of the PH domain and the disordered C‐terminal residues. The information that this void is actually created by the presence of the unresolved residues was not used in our docking calculations and, thus, the fact that the calculated arrangement perfectly fits into this space strongly supports our prediction. It is interesting to note that 3B3J is the only structure in which the N and C termini are located on the same side of the dimer, and in this way differs from any PRMT4 X‐ray structure that is based on truncated versions of the protein, that is, missing the PH domain (PRMT4_28‐140_). In this vein, Yue *et al*. 47 compared PRMT4 without (2V7E) and with cofactor (2V74) and found a different orientation of the N terminus compared to 3B3J (no cofactor). Troffer‐Charlier *et al*. 45 obtained a different arrangement in a second crystal structure of a cofactor‐free protein (3B3G). This evidence suggests that the unique arrangement of the N‐terminal amino acid in 3B3J is significantly induced by the presence of the N‐terminal PH domain and is not solely dependent on the presence of the cofactor. An overview of the location of N and C termini in the deposited PRMT4 crystal structures is depicted in Fig. 5C. Therefore, our findings indicate the cause for the observed structural differences between crystal structures 3B3J (without cofactor, but crystallized with the PH domain) and 3B3G and 2V7E (both crystallized without cofactor and without PH domain).

Based on the docking results of mmPRMT4 comprising the PH domain and the catalytic core, a homology model of ggPRMT4 was generated (Fig. 5D). In our approach, the PH domain and the cofactor‐ and substrate‐binding domains of ggPRMT4 were homology‐modeled independent of each other. The modeled structures were fitted onto the corresponding units of the docking‐derived murine complex using PyMOL and could be placed without violations of their structural integrity, in line with a high structural similarity within the catalytic core domain of the chicken and murine PRMT4 homodimer, yet avian‐specific variations in the PH domain. An analysis of the putative interface between the PH domain and the remaining PRMT4 homodimer revealed that this interface region within the PH domain is more conserved (between *G. gallus* and *M. musculus*) than the rest of the domain. The putative interface in the PH domain is formed by the concavely shaped ß‐sheet at the bottom of the domain (Fig. 5D). Interestingly, the strictly conserved amino acids between the ggPH and mmPH domains mediate inter‐ and intramolecular interactions and are involved in the interface formation, as illustrated in Fig. 5E for the ggPH domain. In contrast, the variable amino acids are located at the surface of the PH domain, thereby potentially accounting for minor species‐specific variations in the interaction domains of PRMT4 binding partners in chicken. The coincidence of the predicted interface with a conserved region of the PH domain supports that the docking‐derived binding mode represents the actual interface.

## Conclusions

Taken together, we identified here the avian ortholog of PRMT4, which reveals more than 90% sequence identity with human PRMT4 and possesses the same substrate specificity toward H3R17 as the other vertebrate homologs. Based on published crystal structures of murine PRMT4 and combined protein–protein docking and homology modeling, we predict a high structural similarity between the mammalian and chicken PRMT4 protein consistent with their overall sequence conservation. Interestingly, our *in silico* structural comparison of the N‐terminal PH domain of chicken and murine PRMT4 identified strictly conserved amino acids that contribute to a newly predicted interaction interface between the PH and the catalytic domain representing the first forecast of their relative spatial arrangement. Furthermore, these findings suggest a structural basis for the recently reported essential functions of the PH domain in substrate recognition and methylation by PRMT4 49. Given the strict transspecies conservation of the amino acids within the PH domain mediating the interaction toward the catalytic core, we propose that targeting this interface with small molecules could be a promising strategy for the design of PRMT4‐selective inhibitors.

## Data accessibility

*Gallus gallus* PRMT4 transcript nucleotide sequence data have been deposited in the GenBank database with accession number KY655811.

## Author contributions

HB performed experiments, analyzed data, and wrote the manuscript. FT, PK, and UMB analyzed data and wrote the manuscript. SR performed experiments and analyzed data. PS analyzed data. CB and MM performed experiments. SP contributed reagents.

## Supporting information


**Fig. S1.** Catalytic activity of mammalian PRMT4.
**Fig. S2.** Mammalian‐specific PRMT4 antibodies recognize recombinant ggPRMT4 protein.
**Fig. S3.** Ramachandran plot of the homology model of ggPRMT4.
**Table S1.** Overview showing all 36 PRMT4 structures deposited in the PDB archive and the structure of the mmPH domain (2OQB).
