# Supplementary material for: Identification and in silico structural analysis of Gallus gallus protein arginine methyltransferase 4 (PRMT4)
Source: FEBS Open Bio. 2017 Oct 10;7(12):1909–23. doi: 10.1002/2211-5463.12323 (PMC5715347; doi:10.1002/2211-5463.12323)
Supplement: Supplementary file 1 — Fig. S1. Catalytic activity of mammalian PRMT4. Fig. S2. Mammalian‐specific PRMT4 antibodies recognize recombinant ggPRMT4 protein. Fig. S3. Ramachandran plot of the homology model of ggPRMT4. Table S1. Overview showing all 36 PRMT4 structures deposited in the PDB archive and the structure of the mmPH domain (2OQB). [file FEB4-7-1909-s001.docx]

**Supporting Information**

**
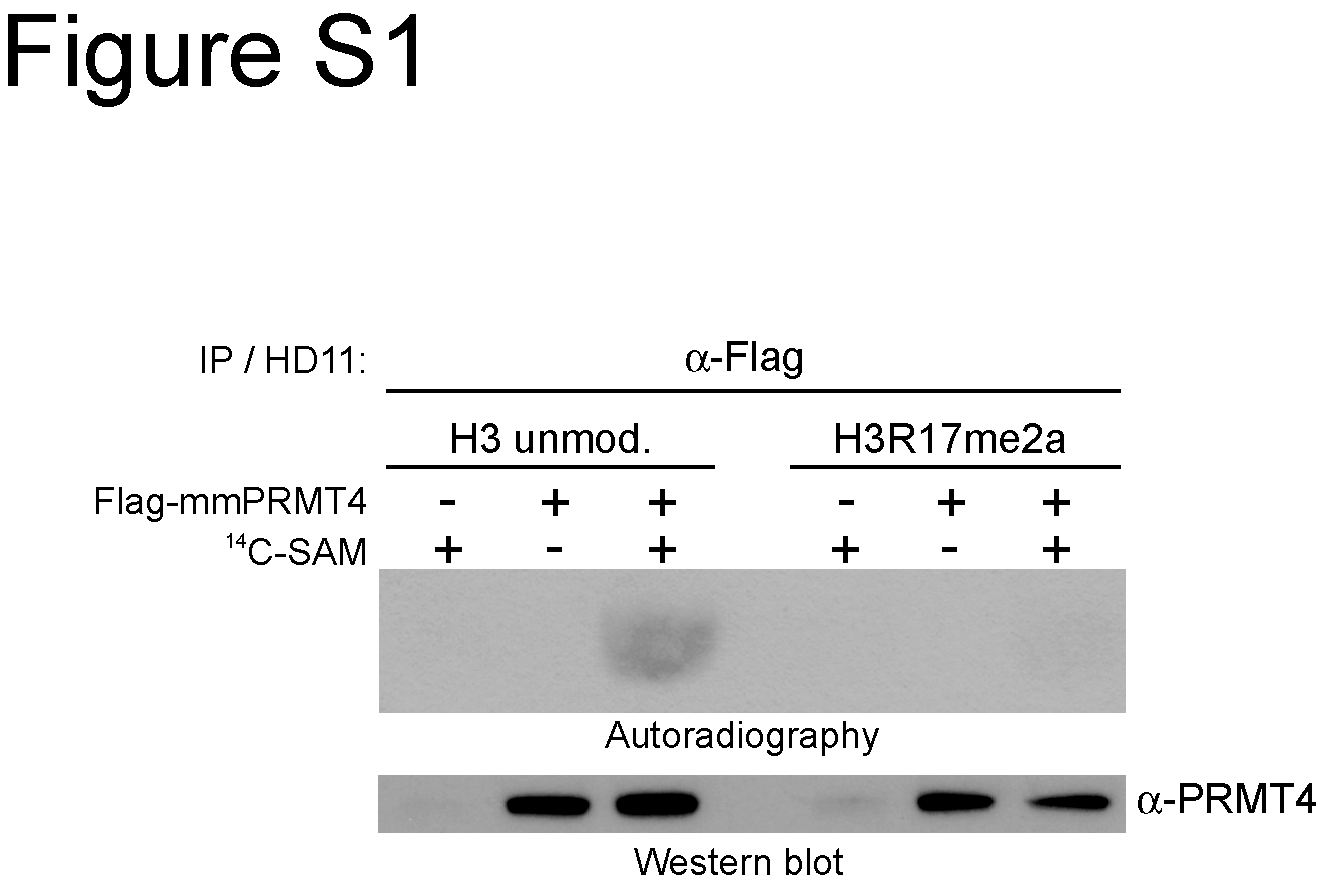
**

**Figure S1:** Catalytic activity of mammalian PRMT4.

As a positive control for the catalytic activity of mammalian PRMT4, Flag-tagged murine PRMT4 (Flag-mmPRMT4) was ectopically expressed (+) or not (-) in HD11 cells. 72 hours post-transfection, cells were lysed and 1 mg of each protein extract was applied to immunoprecipitation (IP) using Flag antibodies (α-Flag). Precipitates were subjected to *in vitro*-methyltransferase (MT) assays (overnight, at 30°C) in the presence of either unmodified or R17me2a-premodified H3 peptides (aa 1-25) and in the absence (-) or presence (+) of ^14^C-labelled SAM. Methylation products were resolved by SDS-PAGE, blotted and analyzed by autoradiography (upper panel). Immunostaining of the blot with PRMT4 antibodies (α-PRMT4) visualizes the bead-bound PRMT4 used in the methylation assay as an input control (lower panel).

**
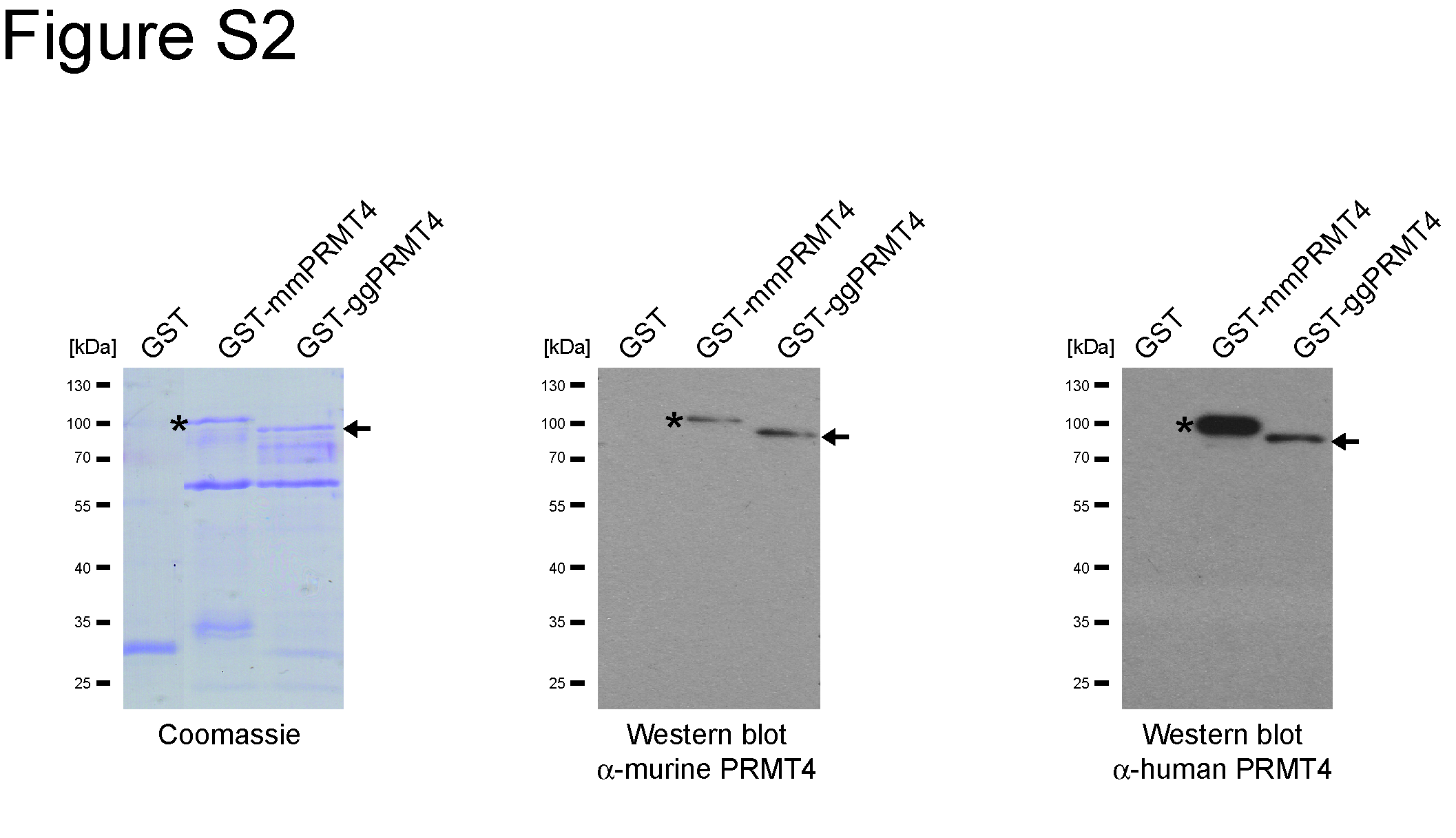
**

**Figure S2:** Mammalian-specific PRMT4 antibodies recognize recombinant ggPRMT4 protein.

GST alone, GST-tagged murine PRMT4 (GST-mmPRMT4) and GST-tagged chicken PRMT4 (GST-ggPRMT4) were expressed in bacteria and purified. Eluted proteins were separated by SDS-PAGE and either visualized by Coomassie blue staining (left panel) or blotted and immunostained using murine (middle panel) or human (right panel) PRMT4 antibodies. The arrows indicate the ggPRMT4 protein. The asterisks mark the mmPRMT4 protein, which possesses a higher molecular weight than the chicken homolog due to its N-terminal extension.

**Table S1**

| PDB code | **Ligand binding site occupied** | **Cofactor binding site occupied** | **N- and C-terminus on one side** | **+ PH domain crystallized** | **PH domain visible** | **Sequence** |
| --- | --- | --- | --- | --- | --- | --- |
| 2V7E | **NO** | **NO** | **NO** | **NO** | **NO** | [145-490](http://www.uniprot.org/blast/?about=Q9WVG6%5B147-490%5D) |
| 2V74 | **NO** | **YES** | **NO** | **NO** | **NO** | [145-490](http://www.uniprot.org/blast/?about=Q9WVG6%5B147-490%5D) |
| 2Y1W | **YES** | **YES** | **NO** | **NO** | **NO** | **135-483** |
| 2Y1X | **YES** | **YES** | **NO** | **NO** | **NO** | **135-483** |
| 3B3F | **NO** | **YES** | **NO** | **NO** | **NO** | [140-480](http://www.uniprot.org/blast/?about=Q4AE70%5B140-480%5D) |
| 3B3G | **NO** | **NO** | **NO** | **NO** | **NO** | [140-480](http://www.uniprot.org/blast/?about=Q4AE70%5B140-480%5D) |
| 3B3J | **NO** | **NO** | **YES** | **YES** | **NO** | [28-507](http://www.uniprot.org/blast/?about=Q4AE70%5B28-507%5D) |
| 4IKP | **NO** | **YES** | **NO** | **NO** | **NO** | [140-480](http://www.uniprot.org/blast/?about=Q86X55%5B140-480%5D) |
| 5DWQ | **YES** | **YES** | **NO** | **NO** | **NO** | [131-479](http://www.uniprot.org/blast/?about=Q86X55%5B134-479%5D) |
| 5DX0 | **YES** | **YES** | **NO** | **NO** | **NO** | [131-479](http://www.uniprot.org/blast/?about=Q86X55%5B134-479%5D) |
| 5DX1 | **YES** | **YES** | **NO** | **NO** | **NO** | [131-479](http://www.uniprot.org/blast/?about=Q86X55%5B134-479%5D) |
| 5DX8 | **YES** | **YES** | **NO** | **NO** | **NO** | [131-479](http://www.uniprot.org/blast/?about=Q86X55%5B134-479%5D) |
| 5DXA | **YES** | **YES** | **NO** | **NO** | **NO** | [131-479](http://www.uniprot.org/blast/?about=Q86X55%5B134-479%5D) |
| 5DXJ | **NO** | **YES** | **NO** | **NO** | **NO** | [131-479](http://www.uniprot.org/blast/?about=Q86X55%5B134-479%5D) |
| 5IH3 | **YES** | **YES** | **NO** | **NO** | **NO** | [127-487](http://www.uniprot.org/blast/?about=Q9WVG6%5B130-487%5D) |
| 5IS6 | **YES** | **YES** | **NO** | **NO** | **NO** | [127-487](http://www.uniprot.org/blast/?about=Q9WVG6%5B130-487%5D) |
| 5IS7 | **YES** | **YES** | **NO** | **NO** | **NO** | [127-487](http://www.uniprot.org/blast/?about=Q9WVG6%5B130-487%5D) |
| 5IS8 | **(YES)** | **YES** | **NO** | **NO** | **NO** | [127-507](http://www.uniprot.org/blast/?about=Q9WVG6%5B130-507%5D) |
| 5IS9 | **YES** | **YES** | **NO** | **NO** | **NO** | [127-487](http://www.uniprot.org/blast/?about=Q9WVG6%5B130-487%5D) |
| 5ISA | **YES** | **YES** | **NO** | **NO** | **NO** | [127-490](http://www.uniprot.org/blast/?about=Q9WVG6%5B130-490%5D) |
| 5ISB | **YES** | **YES** | **NO** | **NO** | **NO** | [127-487](http://www.uniprot.org/blast/?about=Q9WVG6%5B130-487%5D) |
| 5ISC | **NO** | **YES** | **NO** | **NO** | **NO** | [127-487](http://www.uniprot.org/blast/?about=Q9WVG6%5B130-487%5D) |
| 5ISD | **YES** | **YES** | **NO** | **NO** | **NO** | [127-487](http://www.uniprot.org/blast/?about=Q9WVG6%5B130-487%5D) |
| 5ISE | **YES** | **YES** | **NO** | **NO** | **NO** | [127-487](http://www.uniprot.org/blast/?about=Q9WVG6%5B130-487%5D) |
| 5ISF | **YES** | **YES** | **NO** | **NO** | **NO** | [127-487](http://www.uniprot.org/blast/?about=Q9WVG6%5B130-487%5D) |
| 5ISG | **YES** | **YES** | **NO** | **NO** | **NO** | [127-487](http://www.uniprot.org/blast/?about=Q9WVG6%5B130-487%5D) |
| 5ISH | **YES** | **YES** | **NO** | **NO** | **NO** | [127-487](http://www.uniprot.org/blast/?about=Q9WVG6%5B130-487%5D) |
| 5ISI | **(YES)** | **YES** | **NO** | **NO** | **NO** | [127-487](http://www.uniprot.org/blast/?about=Q9WVG6%5B130-487%5D) |
| 5K8V | **YES** | **YES** | **NO** | **NO** | **NO** | [127-487](http://www.uniprot.org/blast/?about=Q9WVG6%5B130-487%5D) |
| 5K8W | **YES** | **YES** | **NO** | **NO** | **NO** | **127-487** |
| 5K8X | **YES** | **YES** | **NO** | **NO** | **NO** | **127-487** |
| 5LGP | **YES** | **YES** | **NO** | **NO** | **NO** | [127-487](http://www.uniprot.org/blast/?about=Q9WVG6%5B130-487%5D) |
| 5LGQ | **YES** | **YES** | **NO** | **NO** | **NO** | [127-487](http://www.uniprot.org/blast/?about=Q9WVG6%5B130-487%5D) |
| 5LGR | **YES** | **YES** | **NO** | **NO** | **NO** | [127-487](http://www.uniprot.org/blast/?about=Q9WVG6%5B130-487%5D) |
| 5LGS | **YES** | **YES** | **NO** | **NO** | **NO** | [127-487](http://www.uniprot.org/blast/?about=Q9WVG6%5B130-487%5D) |
| 5U4X | **YES** | **YES** | **NO** | **NO** | **NO** | **141-481** |
| 2OQB | **--** | **--** | **--** | **YES** | **YES** | [28-140](http://www.uniprot.org/blast/?about=Q4AE70%5B28-140%5D) |

**Table S1:** Overview showing all 36 PRMT4 structures deposited in the PDB archive and the structure of the mmPH domain (2OQB).

**Figure S3**

*In silico* Model Building


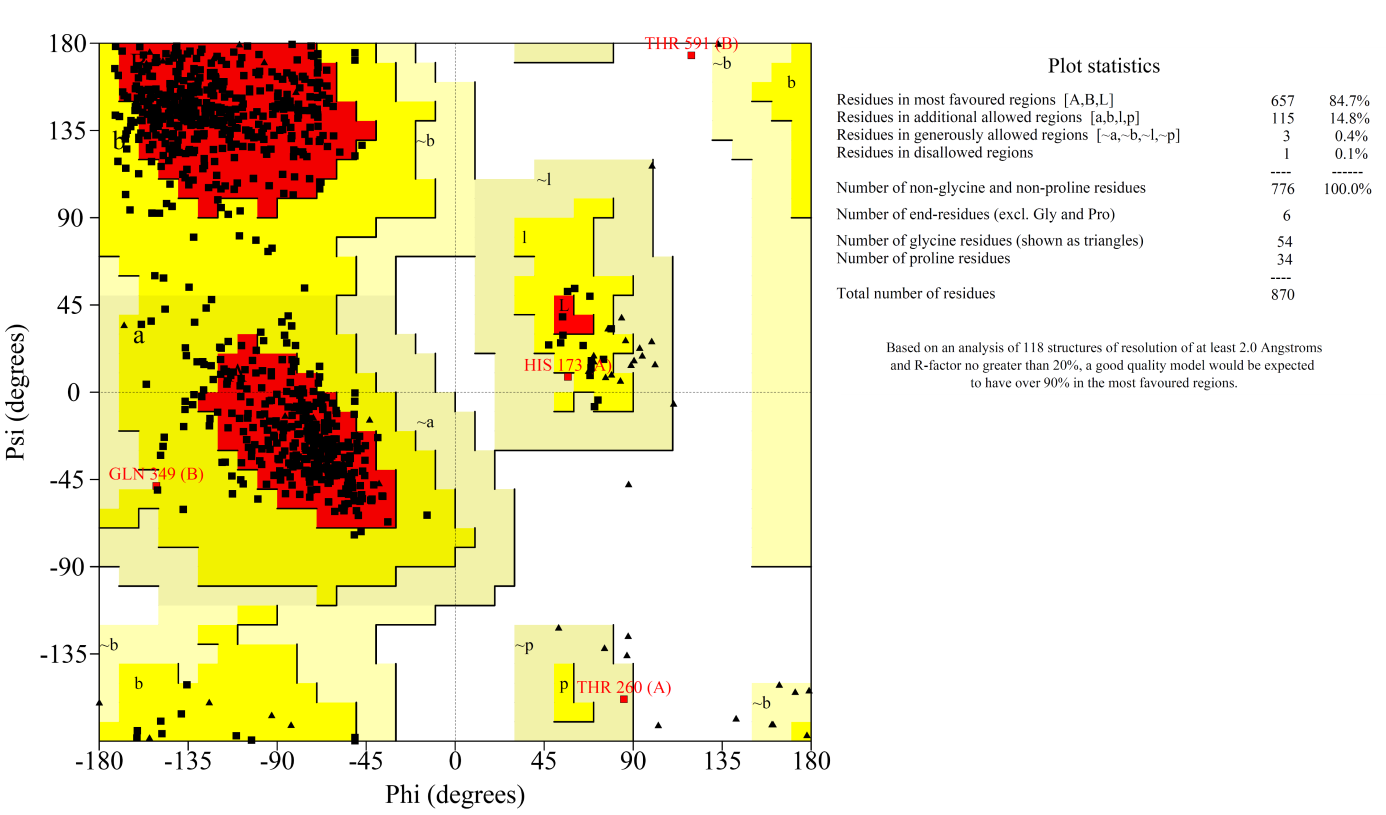


**Figure S3:** Ramachandran plot of the homology model of ggPRMT4. The model contains the homodimer of the cofactor- and substrate-binding domains and the two PH domains. The plot was calculated using the programme PROCHECK [1].

[1] Laskowski RA, MacArthur MW, Moss DS & Thornton JM (1993). PROCHECK - A program to check the stereochemical quality of protein structures. *J. Appl. Crystallogr.* **26,** 283–291.
